# Supplementary material for: Clinical and growth outcomes after meconium-related ileus improved with Gastrografin enema in very low birth weight infants
Source: PLoS One. 2022 Aug 11;17(8):e0272915. doi: 10.1371/journal.pone.0272915 (PMC9371358; doi:10.1371/journal.pone.0272915)
Supplement: Attachment — Submitted filename: Plos MRI review.docx [file pone.0272915.s004.docx]

- Please concise the previous studies related to meconium related ileus if possible in the introduction section.
- We define the primary aim and secondary aims more clearly in the hypothesis section.
- Can we combine the table 1 and 2. Keep the maternal factors first followed by infant factors.
- Can we look into the factors why there is delay in start of the feeds after in gastrograffin group?
- I would advised to keep the hypothesis focused to the characteristics of MRI and immediate outcomes
- I would take the growth outcomes out of the hypothesis.
- Can we change the MRI to meconium associated ileus.
- The BPD effect is more related to gestation age rather than MRI. I would not include that in the conclusion.
