# Supplementary material for: Clinical and growth outcomes after meconium-related ileus improved with Gastrografin enema in very low birth weight infants
Source: PLoS One. 2022 Aug 11;17(8):e0272915. doi: 10.1371/journal.pone.0272915 (PMC9371358; doi:10.1371/journal.pone.0272915)
Supplement: Attachment — Submitted filename: Response to reviewers_3.docx [file pone.0272915.s005.docx]

We deeply appreciate your valuable and kindest comments.

Reviewer 1.

Thank you so much for your endorsement of our paper.

Reviewer 2.

Thank you for your precious comments for taking the time to point out options to improve our paper. We have revised the manuscript following your suggestions as follows.

1. Please concise the previous studies related to meconium related ileus if possible in the introduction section.

: Thank you for your helpful comments. We’ve made concise the previous studies related to meconium related ileus in the introduction section. We’ve highlighted the sentences for you to check.

2. We define the primary aim and secondary aims more clearly in the hypothesis section.

: Thank you for your helpful comments.

We’ve changed the sentence of aims in the background of the abstract and in the introduction of the main manuscript with your comments. We’ve highlighted the sentences for you to check.

3. Can we combine the table 1 and 2. Keep the maternal factors first followed by infant factors.

: Thank you for your helpful comments.

We’ve combined table 1 and 2 into table 1 with keeping the maternal factors first followed by infant factors. We’ve highlighted the sentences for you to check.

4. Can we look into the factors why there is delay in start of the feeds after in gastrografin group?

: Thank you for your helpful comments. We added the reasons of the delay in the start of the feeding in discussion. We’ve highlighted the sentence for you to check.

5. I would advised to keep the hypothesis focused to the characteristics of MRI and immediate outcomes.
6. I would take the growth outcomes out of the hypothesis.

: Thank you for your sincere comments (5.6).

To the best of our knowledge, the current study is the first to report growth outcomes after resolved MRI by GaE treatment. We propose this is the merit of our study. Therefore, we are sorry that we cannot take out the growth outcome. I hope for your understanding and consideration.

7. Can we change the MRI to meconium associated ileus.

: Thank you for your sincere comments. In 1999, Kubota et al. proposed that the term meconium-related ileus (MRI) should include meconium plug syndrome and meconium disease because both diseases have speculated similar pathogenesis [6]. We propose MRI is the most appropriate diagnosis for enrolled patients in our study. Therefore, we are sorry that we cannot change the MRI to meconium associated ileus. I hope for your understanding and consideration.

8. The BPD effect is more related to gestation age rather than MRI. I would not include that in the conclusion.

: Thank you for your helpful comments. We’ve deleted the BPD effect in conclusion of the abstract and the main manuscript.
